# SARS-CoV-2-Specific T Lymphocytes Analysis in mRNA-Vaccinated Patients with B-Cell Lymphoid Malignancies on Active Treatment

**DOI:** 10.3390/vaccines12090961

**Published:** 2024-08-26

**Authors:** Patricia García Ramírez, Marta Callejas Charavia, Raquel Oliva Martin, Ana María Gómez La Hoz, Miguel Ángel Ortega, Julio García Suárez, Melchor Álvarez-Mon, Jorge Monserrat Sanz

**Affiliations:** 1 Hematology Department, University Hospital “Príncipe de Asturias”, Alcalá de Henares, 28805 Madrid, Spain; marta.callejascha@salud.madrid.org; 2 Department of Medicine, University of Alcalá, Alcalá de Henares, 28871 Madrid, Spain; raquel_1617@hotmail.com (R.O.M.); alahoz1199@gmail.com (A.M.G.L.H.); miguel.angel.ortega92@gmail.com (M.Á.O.); mademons@gmail.com (M.Á.-M.); jorge.monserrat@uah.es (J.M.S.); 3 IRYCIS Unit (Instituto Ramón y Cajal de Investigación Sanitaria), 28034 Madrid, Spain

**Keywords:** vaccine, SARS-CoV-2, antibodies, CD4^+^ and CD8^+^ T lymphocytes, B-cell lymphoid malignancy

## Abstract

Background: Patients with B-lymphocyte malignancies (BCMs) receiving B-lymphocyte-targeted therapies have increased risk of severe COVID-19 outcomes and impaired antibody response to SARS-CoV-2 mRNA vaccination in comparison to non-hematologic oncologic patients or general population. Consequently, it is vital to explore vaccine-induced T-lymphocyte responses in patients referred for the understanding of immune protection against SARS-CoV2 infections. The objective of the present study was to analyze the recall immune responses carried out by T lymphocytes after two COVID-19 mRNA vaccine doses. Methods: We enrolled 40 patients with BCMs and 10 healthy controls (HCs) after 4 weeks from the second mRNA vaccine dose. Spike (S)-specific T-lymphocyte responses were assessed in peripheral blood mononuclear lymphocytes (PBMCs) by intracellular IFN-γ staining combined with flow cytometry. Furthermore, the humoral response was assessed with the measurement of anti-spike antibodies. Results: From March to July 2021, 40 patients (median age 68) received mRNA vaccines. The overall antibody response for BCMs was 52.5% versus 100% for the healthy controls (*p* = 0.008). The antibody response was different across BCMs: 18.75% for non-Hodgkin lymphoma, 54.5% for chronic lymphocytic leukemia, and 92.3% for multiple myeloma. Responses varied by malignancy type and treatment, with anti-CD20 therapies showing the lowest response (6.7%). T-lymphocyte analysis revealed reduced numbers and altered differentiation stages in patients compared to the controls. However, the vaccine-induced T response was generally robust, with variations in specific T subpopulations. Conclusions: mRNA vaccines induced significant humoral and cellular immune responses in B-cell lymphoid malignancy patients, although responses varied by treatment type and malignancy. Further research is needed to optimize vaccination strategies in this population.

## 1. Introduction

Patients with mature B-lymphocyte malignancies (BCMs) undergoing active treatment with B-lymphocyte targeting therapies, such as anti-CD20 mAb, BTK inhibitor therapy, and chimeric antigen receptor T-lymphocyte therapy (CAR-T), are at higher risk for severe COVID-19 and death compared to the general population and people with other cancers [1,2,3,4]. This is true even when following standard messenger RNA (mRNA) vaccination regimens including booster or additional vaccine doses [5,6].

It has been demonstrated that neutralizing antibody responses after vaccination are highly predictive of immune protection from developing severe COVID-19 following SARS-CoV-2 infection [7,8,9]. In healthy subjects, mRNA vaccines induce robust humoral responses in most people. However, in patients with mature BCMs receiving B-lymphocyte targeting agents, only a small subset (less than 23%) of patients achieves serological conversion after complete vaccination [10,11,12,13] compared to 79–98% in patients with solid tumors. Poor responses are particularly pronounced in patients with chronic lymphocytic leukemia and non-Hodgkin lymphomas over the span of 6 months after treatment with anti-CD20 antibodies [14,15,16].

It has been demonstrated that exposure to SARS-CoV-2 can generate strong virus-specific T-lymphocyte responses despite the absence of seroconversion, and these robust cellular responses are associated with mild COVID-19 disease [17]. In addition to antibody responses, the development of antiviral CD4^+^ and CD8^+^ T lymphocytes has been shown to improve survival in patients with COVID-19 and hematologic cancers [18]. Vaccine-induced memory T lymphocytes are essential for providing help to B lymphocytes for antibody production as well as aiding in viral clearance upon subsequent exposure. While the effect of SARS-CoV-2 mRNA vaccination on the antibody immune response in BCM patients receiving B-lymphocyte-targeted therapies have largely been described, their impact on the long-lived T-lymphocyte responses in in this specific patient population have not yet been fully understood. The few conducted studies in this area have been small series, and the majority of them without active treatment. They evaluated the SARS-CoV-2–specific effector and memory CD4^+^ and CD8^+^ T cell responses after mRNA vaccination [19,20,21], which plays a pivotal role in understanding the vaccine-induced immunity in BCMs. To evaluate the SARS-CoV-2–specific T lymphocyte responses after vaccination, it is necessary to accurately evaluate the T-lymphocyte activation/differentiation stage subsets including naive (T_N_, CD45RA^+^CCR7^+^), central memory (T_CM_, CD45RA^−^CCR7^+^), effector memory (T_EM_, CD45RA^−^CCR7^−^), and effector (T_E_, CD45RA^+^CCR7^−^ T cells.

In the present study, we performed an integrated analysis of B-lymphocyte and T-lymphocyte immune responses in BCM patients on active therapy after two mRNA vaccine doses. To the best of our knowledge, this is the first to investigate the recall immune responses carried out by both CD4+ and CD8+ TCM and TEM lymphocytes in this type of patient in comparison with healthy controls. We particularly focused our analysis on a unique population of BCM patients, such as B-cell non-Hodgkin lymphoma (B-NHL), multiple myeloma (MM), and chronic lymphocytic leukemia (B-CLL), due to their severe immune dysregulation and impairment of all immune system components.

## 2. Materials and Methods

### 2.1. Study Design and Patients

In the current single-center, prospective, observational, and real-world cohort study, we investigated the efficacy of mRNA COVID-19 vaccines in 40 patients with BCMs, diagnosed and followed up at the Príncipe de Asturias University Hospital, who were vaccinated against SARS-CoV-2 as part of the Spanish national vaccination program. Ten healthy volunteers from the community, aged >18 years and without known previous SARS-CoV-2 infection, who had received two consecutive BNT162b2 COVID-19 vaccine doses, served as the healthy controls (HCs). On 15 March 2021, a COVID-19 vaccination program began in Spain. At that time, the Alpha variant (B.1.1.7) was dominant across large parts of the world including Spain, until the Delta variant (B.1.617.2) became the dominant variant of the disease worldwide in July 2021.

Eligible patients were 18 years or older with BCMs including B-NHL, MM, and B-CLL and were undergoing active systemic anti-cancer treatment. Active anti-cancer therapy was defined as current B-lymphocyte-directed therapies or any anti-cancer therapy within 6 months before the first vaccine dose. To obtain a real-world representation of the B-lymphocyte lymphoid malignancy population, patients with prior COVID-19 were not excluded. According to the department’s policy, patients were vaccinated 7 to 14 days after their most recent course of chemo-immunotherapy/immunotherapy. During the study period, due to the high incidence of COVID-19 in our community, we screened all asymptomatic patients for COVID-19 RNA before every cycle of outpatient or inpatient antineoplastic therapy. Information regarding the demographic details, disease characteristics, cancer treatment, COVID-19 infection status, and SARS-CoV-2 vaccination details were collected between April and December of 2021 from the electronic medical records. Participants with B-lymphocyte lymphoid malignancies with evidence of a positive SARS-CoV-2 PCR test were considered as breakthrough infection.

The principal exclusion criteria for patients and HCs included the presence of: (i) autoimmune disorders or active non-hematological malignant disease; (ii) HIV or active hepatitis B and C infection; and (iii) end-stage renal disease. These disease entities could confound the effect on Ab response following vaccination.

The first visit of the patient groups was before the first vaccine dose to analyze the basal immunologic condition; a complete blood count test and the total serum immunoglobulin levels were determined. In the HC group, the first contact was a telephone call to subjects vaccinated recently at our center to offer the participation in this study. In both groups, at the second visit, venous blood was obtained 4 weeks after the second dose of the mRNA-1273 or BNT162b2 vaccine administration to evaluate the immune humoral and cellular responses.

Samples were stored at 4 °C until processing at the Laboratory of Immune System Disease of the Department of Medicine and Medical Specialties of the University of Alcalá, within a period not exceeding one hour. The data collection cutoff date was December 2021.

The study was approved by the Institutional Review Board (IRB) of the Príncipe de Asturias University Hospital (LIB 15/2021) and was conducted as per the ethical principles of the Declaration of Helsinki and the International Conference on Harmonization Good Clinical Practice guidelines. All controls and patients provided written informed consent prior to enrollment in our study.

### 2.2. Anti-SARS-CoV-2 Antibody Assays

IgG antibodies against the receptor-binding domain (S-RBD IgG) were assessed at the Dept. of Microbiology, Príncipe de Asturias University Hospital, by using a chemiluminescence immunoassay (CLIA) through an orthogonal algorithm: the first step of the algorithm consisted of a screening test to detect the total antibodies (IgM + IgG) against the Spike (S) protein (RBD region of the S1 subunit) (SARS-CoV-2 total COV2T [Siemens, Erlagen, Germany]). The system reports results as non-reactive (index < 1) or reactive (index ≥ 1.0), the latter being considered positive for IgG antibodies to SARS-CoV-2 with a sensitivity of 100% and a specificity of 99.95%. Positive samples for the total antibodies were subjected to a second semiquantitative test to detect IgG antibodies using a CLIA assay that detected antibodies against the S1 proteins (SARS-CoV-2 IgG COV2G [Siemens, Erlagen, Germany). Antibody levels were normalized according to the WHO standard, and the results were reported as SARS-CoV-2 binding antibody units per milliliter (1 unit of our local lab equals 21.80 BAU/mL).

### 2.3. Lymphocyte Profiling and SARS-CoV-2-Specific Memory CD4^+^ and CD8^+^ T Lymphocyte Responses

Fresh PB mononuclear lymphocytes (PBMCs) were separated by centrifugation in a density gradient with Hypaque-Ficoll^®^ (Sigma Aldrich, Merk, Germany). Then, lymphocytes were placed in RPMI1640 medium (Biowhittaker, Verviers, Belgium, Europe) with the addition of ten percent fetal-calf-serum (GIBCO), 25 mM HEPES buffer (Biowhittaker-Products), and one percent penicillin/streptomycin antibiotic (Biowhittaker-Products). Lymphocyte counting was performed by a microscope using a counter chamber, following the blue colorimetric dead exclusion lymphocytes. Finally, the PBMCs were adjusted to 1 × 10^6^ lymphocytes/mL. T lymphocytes were phenotypically analyzed by flow cytometry to study the activation subsets of CD4^+^ and CD8^+^ T lymphocytes after incubation with the next surface labeled monoclonal antibodies (mAbs): CCR7-PeCy7 (Phycoerythrincyanine-7, Becton-Dickinson (BD), San Diego CA, USA), CD8-A405 (Allophycocyanin-Alexa405, Invitrogen, Carlsbad CA, USA), CD45RA-APC (Allophycocyanin, BD), CD3-A700 (Allophycocyanin Alexa 700, BD), and CD4-PerCP (Peridininchlorophyll protein, BD). Data were acquired on the FACSAriaII SORP (BD) and analyzed using BD-FACSDiva 6.0 software.

SARS-CoV-2-specific memory T-lymphocyte responses to the spike protein and RBD peptide pool were assessed using intracellular IFNγ staining combined with flow cytometry. It has the specific advantage of enabling the simultaneous assessment of multiple phenotypic, differentiation, and functional parameters pertaining to responding T lymphocytes. These attributes make the technique particularly suitable for the assessment of T-lymphocyte immune responses induced by novel vaccines. To characterize IFNγ-producing S-specific T lymphocytes and to understand whether vaccine-induced T lymphocytes display features of longevity, we studied the lymphocyte frequencies and total lymphocyte numbers of S-specific T lymphocytes among the differentiation subpopulations of CD4^+^ and CD8^+^ T lymphocytes. We defined T-lymphocyte activation/differentiation stage subsets in both the CD4 and CD8 T lymphocytes, attending to CD45RA+ and CCR7+ antigen surface expression in naive (T_N_, CD45RA+CCR7+), central memory (T_CM_, CD45RA-CCR7+), effector memory (T_EM_, CD45RA-CCR7-), and effector (T_E_, CD45RA+CCR7-) T cells. Briefly, 2 × 10^6^ PBMCs were incubated during 6 h at 37 °C and 5% of CO_2_ atmosphere under two different conditions. (i) Peptide pool tube including lymphocytes, 10 μg/mL brefeldin from *Penicillium brefeldinanum* (Sigma-Aldrich, Merck, Darmstadt, Germany), and the Spike protein of the SARS-CoV-2 Wuhan wild type using PepTivator^®^ Peptide Pools; this is a pool of lyophilized peptides (Prot_S1 6 nmol/peptide) covering the N-terminal S1 domain of the surface glycoprotein (“S”) of SARS-CoV2; Prot_S 6 nmol/peptide complete covering selected immunodominant sequence domains of the spike protein; and Prot_S+ 6 nmol/peptide covering the C-terminal S2 domain (from Miltenyi Biotec). Therefore, this tube can provide insights into induced IFNγ production because stimulation of antigen-specific T lymphocytes with peptide pools causes the secretion of effector cytokines, which then allow for the detection and isolation of antigen-specific T lymphocytes. (ii) Positive control tube including lymphocytes, 10 μg/mL brefeldin, 50 ng/mL phorbol-12-myristate 13-acetate (PMA P1585, Sigma Aldrich, Merck, Darmstadt, Germany), and 100 μg/mL ionomycin from *Streptomyces conglobatus* (Sigma Aldrich, Merck, Darmstadt, Germany); this tube provides insights into the induced production of IFNγ. The negative control tube includes lymphocytes and 10 μg/mL brefeldin. This tube provides insights into spontaneous IFNγ production and was the control for the stimulation with the peptide pool and PMA.

After stimulation, the PBMCs were washed and incubated for 20 min at 4 °C with the following surface-labeled mAbs: anti-CD3-PerCP (Peridinin chlorophyll protein, Becton-Dickinson, San Diego, CA, USA), anti-CD8-AlexaFluor405 (Allophycocyanin Alexa 405, Invitrogen, Carlsbad, CA, USA), anti-CD45RA-APC (Allophycocyanin, Becton-Dickinson, San Diego, CA, USA), anti-CCR7-PeCy7 (Phycoerythrin-cyanine 7, Becton-Dickinson, San Diego, CA, USA), anti-CD27-APC-AlexaFluor780 (Allophycocyanin, eBioscience, San Diego, CA, USA), and a dead lymphocyte discriminator LIVE/DEAD^TM^ Fixable Aqua Dead Lymphocyte Stain Kit with a 405 nm excitation (Invitrogen, Carlsbad, CA, USA). For the intracellular assay, lymphocytes were treated with the FIX & PERM^®^ Lymphocyte Fixation and Permeabilization Kits (Invitrogen, San Francisco, CA, USA), and IFNγ was stained with anti-IFNγ-A700 (Allophycocyanin Alexa 700, BD). Samples were acquired on the FACSAria II SORP (BD) and analyzed using BD-FACSDiva 6.0 software. For the peptide pool tube, we acquired at least 1.5 million PBMCs.

Vaccine-induced T-cell immune memory responses were classified according to the frequencies of IFN-γ-producing S-specific T memory generated in response to peptide stimulation as follows: high responders (HR, ≥upper limit of reference range in HCs), normal responders (NR, HCs-based reference intervals), and low responders (LR, <lower limit of the reference range in HCs).

### 2.4. Statistical Analysis

Analysis was performed using IBM SPSS Statistics 23.0 software (Statistical Package for the Social Sciences, version 23.0, Armonk, NY, USA). Since most variables did not fulfill the normality hypothesis, the Mann–Whitney U-test for non-parametric data was used to analyze the differences between groups, and the Pearson correlation coefficient was used for the association between indicated continuous variables. All statistical tests were conducted with a 95% confidence interval and a *p*-value of <0.05. Results were shown as a box plot representing the median and the 25th and 75th quartiles.

The absolute number of circulating T-lymphocytes subset were calculated by the percentage of each subpopulation in the PBMCs determined by flow cytometry multiplied by the total number of lymphocytes per microliter measured by Beckman Coulter, Inc. (Brea, CA, USA).

## 3. Results

### 3.1. Patient Characteristics

From 20 March 2021 to 21 July 2021, 40 patients (median age 68 years [IQR 61–72 years], 67.5% male) with BCMs received two doses of an mRNA vaccine and were included in the analysis. Thirty-eight received mRNA-1273 (Moderna) and BNT162b2 (BioNtech/Pfizer). All ten HCs (59 years, 60% male) received two doses of BNT162b2 in the same period and were also included in the study. The median time between the first and second doses was 28 days (IQR 27–30 days). The median interval between the second vaccination and sample collection was 39 days (IQR 22–30 days) in the patient cohort and 22 days (IQR 21–31 days) in the HCs. The study flowchart in Supplementary Materials Figure S1 shows the process of selecting the patients for the analysis of mRNA vaccination.

Sixteen (40%) patients were diagnosed with B-NHL, 13 (32.5%) with MM, and 11 (27.5%) with B-CLL. Fifteen (37.5%) patients were receiving anti-CD20 alone or with chemotherapy, 11 (27.5%) BTK inhibitors, 7 (17.5%) anti-CD38 in monotherapy or associated with proteasome inhibitors (PI) or IMIDs, 5 (12.5%) with immunomodulatory drugs (IMIDs) alone, and 1 (2.5%) with other target therapies such as venetoclax 1 (2.5%) or VCD (bortezomib, cyclophosphamide, dexamethasone). Thirty-five patients (87.5%) were actively treated, and five patients (12.5%) had completed anti-CD10 + chemotherapy treatment in the last 6 months. The median interval between the last anti-CD20 based therapy and study assessment was 40 days (range: 28–163). Most patients (97.5%) had good control of their hematologic disease at the moment of vaccination, with 21 of them (52.5%) in complete response, 18 in partial response (45%), and 1 in progression. At the time of vaccination, 13 patients (32.5%) had lymphopenia (absolute lymphocyte count <1.0 × 10^9^/L), 31 (77.5%) had IgM low basal levels (<50 mg/dL), and 21 patients (52.5%) had IgG low levels (<700 mg/dL). The median Ig G level in the B-NHL patients was 790.5 mg/dL (IQR: 629–969), 665 mg/dL (IQR: 568–1235) in the CLL cohort and 485 mg/dl (IQR: 443–614) in the MM group. The main baseline demographic and clinical characteristics are summarized in Table 1.

### 3.2. Humoral Response to mRNA Vaccines

Eight (20%) patients had previous SARS-CoV-2 infection, and six of them had detectable antibodies at the baseline. None of the HCs had previously known SARS-CoV-2 infection. The anti-S1 IgG antibody concentration of hybrid immunity (previous infection and two doses of mRNA vaccine) in patients with B-lymphocyte lymphoid malignancies was higher than in the patients who had not had a previous infection. A total of 83.3% with hybrid immunity had > 3270 BAU/mL.

Overall, 52.5% of patients (21/40) showed an antibody response in comparison to HCs (10/10, 100%). Using Fisher’s exact test, given the sample size, we obtained a statistically significant difference (*p* = 0.008). The BCMs associated with the lowest response were B-NHL (3/16, 18.75%), followed by CLL (6/11, 54.54%), and MM (12/13, 92.3%). When the humoral response was evaluated by treatment, we detected a significantly impaired humoral response in patients receiving anti-CD20 Ab-based treatments with an antibody response rate of only 6.7% (1/15). Patients treated with IMID monotherapy showed the highest humoral response rate of all active therapies with 100% (5/5) of seroconversion. In CLL patients treated with BTK inhibitors, the humoral response was 63.6% (7/11).

### 3.3. Phenotypical Characterization of CD4^+^ and CD8^+^ T Lymphocytes and Their Activation/Differentiation Stage Subpopulations

**Figure 1:**
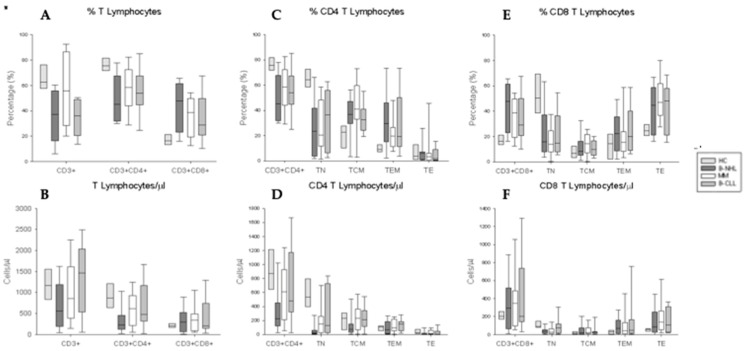
Phenotypic characterization of T and their CD4^+^ and CD8^+^ in all cohorts. (**A**) Box plot represents the percentage of T. (**C**,**E**) Box plots represent the percentage of CD4^+^ and CD8^+^ T and their different activation subsets. (**B**) Box plot represents the numbers of T. (**D**,**F**) Box plots represent the numbers of CD4^+^ and CD8^+^ T and their different activation subsets. HCs, B-NHL, MM, B-CLL.

First, to obtain a global view of the phenotypic landscape of T-lymphocyte compartment in the PBMCs, we studied the distribution and numbers of CD4^+^ and CD8^+^ T lymphocytes and their T_N_, T_CM_, T_EM_, and T_E_ activation subsets in patients with B-NHL, MM, or CLL with respect to HCs after two administrations of SARS-CoV-2 vaccination. Our analysis revealed a significant reduction in the T lymphocyte percentage and numbers in patients with B-NHL, and in the percentage of patients with CLL with respect to HCs; however, the T-lymphocyte levels in patients with MM were normal. We also found a redistribution with a significant decrease in CD4^+^ T lymphocytes associated with an increase in CD8^+^ T lymphocytes in all three BCM cohorts. Finally, only patients diagnosed with B-NHL showed a significant decrease in the CD4^+^ T lymphocyte levels (Figure 1A,B).

Regarding to distinct T-lymphocyte activation subsets, we observed a significant reduction in CD4^+^ T_N_ lymphocyte percentages in the B-NHL and B-CLL patients, associated with a significant increase in the CD4^+^ T_CM_ and T_EM_ subsets, and in MM patients associated with a significant increase only in the CD4^+^T_CM_ with respect to HCs. Nevertheless, the reduction in the CD4^+^ T lymphocyte numbers in B-NHL patients was only due to the CD4^+^ T_N_ subset; also, MM patients showed a reduction in the numbers of the CD4^+^ T_N_ subset (Figure 1C,D). On the other hand, we also studied the percentage and numbers of the activation subsets in CD8^+^T lymphocytes from all groups of pathologies and HCs. We found a significant reduction in CD8^+^T_N_ lymphocyte percentage and numbers in the B-NHL and MM patients, and only in the percentage of B-CLL patients with respect to HCs (Figure 1E,F).

### 3.4. S-Specific IFNγ T Lymphocyte Responses Induced by mRNA Vaccination

In both populations of SARS-CoV-2 vaccinated BCMs and HC subjects, we analyzed the frequencies and numbers of S-specific IFNγ-producing lymphocytes at the different activation subsets of CD4^+^ and CD8^+^ T lymphocytes to identify immune features associated with superior recall function and longevity. Concretely, we explored the ability of the T_CM_ and T_EM_ CD4^+^ and CD8^+^ lymphocyte subpopulations to produce IFNγ after six hours of stimulation with the peptide virus pool or PMA in patients with B-NHL, MM, CLL, and HCs. Representative dot plots of IFNγ-secreting CD4^+^ and CD8^+^ T lymphocytes with S-specific pool stimulation are shown in Supplementary Material Figure S2. No significant differences were found in either the percentage or number of IFNγ secreting T_CM_ and T_EM_ CD4^+^ and CD8^+^ lymphocytes between the three groups of patients and HCs (Supplementary Material Figure S3), although there were heterogeneous results among the 40 BMC patients. Interestingly, three B-NHL (18.75%) patients, and one MM (7.7%) patient did not show positive IFNγ^+^ production by T_CM_ CD4^+^ lymphocytes. All HCs showed IFNγ+ CD4+ and CD8+ T lymphocyte responses. Importantly, the type of cancer treatment including anti-CD20 agents and BTKi did not affect the S-specific memory CD4^+^ and CD8^+^ T lymphocyte response.

Additionally, stimulation with PMA was used to confirm the capacity of T lymphocytes to produce effector cytokines and served as the positive control for the flow cytometry assay. Representative dot plots of IFNγ-secreting memory CD4^+^ and CD8^+^ T lymphocytes following PMA stimulation are shown in Supplementary Material Figure S4. We found a significant increase in the percentage of IFNγ secretion from the T_CM_ subpopulation of CD4^+^ and CD8^+^ T lymphocytes in patients with CLL compared to the HCs. In terms of numbers, we also found a relevant increase in the levels of IFNγ by T_EM_ CD8^+^ T lymphocytes in B-CLL patients with respect to the HCs (Supplementary Material Figure S5).

**Figure 2:**
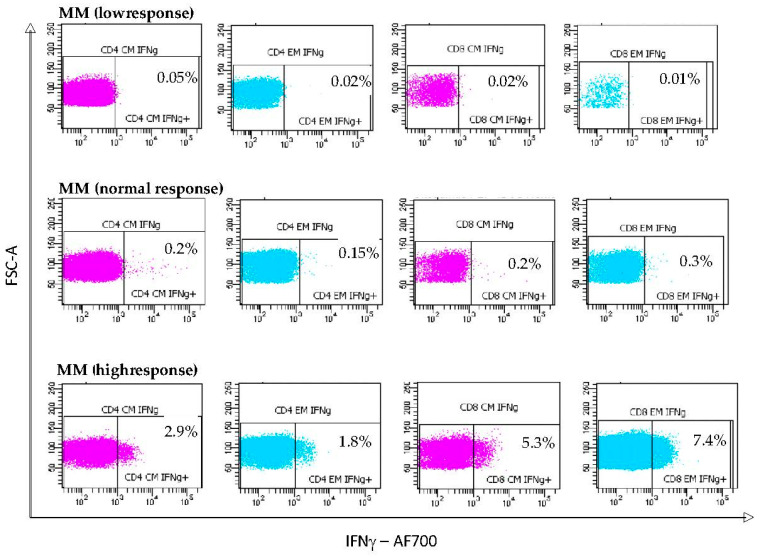
Dot plots represent the percentage of intralymphocyteular IFNγ secretion from T_CM_ and T_EM_ subpopulations of CD4^+^ and CD8^+^ T after stimulation with the peptide pool in MM patients who generated a low, normal, and high response, respectively.

Next, we decided to deepen the analysis of the responses of the different individuals in each of the three groups of BCM patients. For each T_CM_ and T_EM_ CD4^+^ and CD8^+^ T lymphocyte subset, and according to the IFNγ expression after S-specific pool stimulation, we classified patients as high responders (HRs ≥ upper limit of reference range in HCs), normal responders (NRs, HCs-based reference intervals), and low responders (LRs, <lower limit of the reference range in HCs). Representative dot plots of secreting IFNγ CD4^+^ and CD8^+^ T lymphocytes with S-specific pool stimulation in patients with MM (Figure 2).

**Figure 3:**
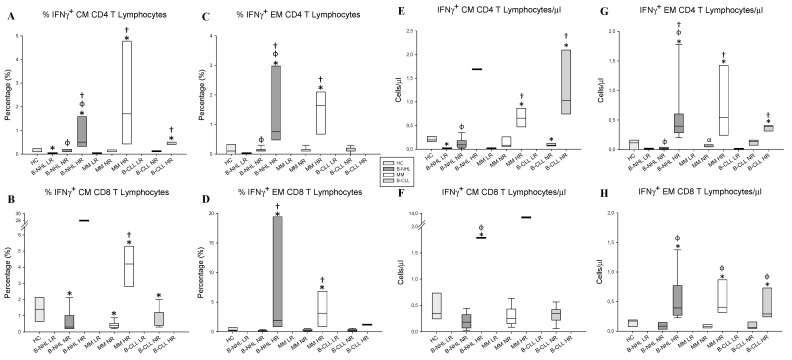
Percentage and numbers of intralymphocyteular IFNγ secretion from T_CM_ and T_EM_ subpopulations of CD4^+^ and CD8^+^ T after stimulation with the peptide pool. (**A**,**B**) Box plots represent the percentage of IFNγ production by the T_CM_ subpopulations of CD4^+^ and CD8^+^ T. (**C**,**D**) Box plots represent the percentage of IFNγ production by the T_EM_ subpopulations of CD4^+^ and CD8^+^ T lymphocytes. (**E**,**F**) Box plots represent the numbers of IFNγ production by the T_CM_ subpopulation of CD4^+^ and CD8^+^ T. (**G**,**H**) Box plots represent the numbers of IFNγ production by the T_EM_ subpopulation of CD4^+^ and CD8^+^ T lymphocytes. HCs, B-NHL, MM, B-CLL lymphocytes. * *p* < 0.05 HCs vs. patients; Φ *p* < 0.05 LR vs. NR vs. HR; † *p* < 0.05 NR vs. HR; α *p* < 0.05 B-NHL vs. MM vs. B-CLL; β *p* < 0.05 MM vs. B-CLL.

Twenty-five percent (4/16) of patients with B-NHL, 30.76% (4/13) of patients with MM, and 27.27% (3/11) of patients with CLL were IFNγ-producing T_CM_ CD4^+^ T lymphocyte HRs with values significantly higher than those observed in the HCs. However, 18.75% (3/16) of the B-NHL patients were LRs (Figure 3A). Similar results were obtained when studying the HR response of the IFNγ-producing T_EM_ subpopulation of CD4^+^ T lymphocytes in patients with B-NHL and MM; however, the CLL patients did not show a HR (Figure 3C).

Regarding the percentage of IFNγ-producing T_CM_ subpopulation of CD8^+^ T lymphocytes, only 23.07% (3/13) of MM patients were HRs. Furthermore, 93.75% (15/16) of B-NHL patients and 100% (11/11) of B-CLL patients were NRs. (Figure 3B). Focusing on the IFNγ-producing T_EM_ subpopulation of CD8^+^ T lymphocytes, 25% (4/16) of B-NHL patients and 30.76% (4/13) of patients with MM that presented were HRs (Figure 3D). No patients in any subgroup were LRs.

When investigating the numbers of each subgroup, we support that only three patients with B-NHL were LRs with respect to numbers of the IFNγ-producing T_CM_ subpopulation of CD4^+^ T lymphocytes (Figure 3E). Focusing on the numbers of the IFNγ-producing T_CM_ or T_EM_ CD8^+^ T lymphocytes, we did not find LRs (Figure 3F,H).

In parallel, in the three groups of pathologies and HCs, we studied IFNγ expression by T_CM_ or T_EM_ CD4^+^ and CD8^+^ after PMA stimulation. Representative dot plots of secreting IFNγ T_CM_ or T_EM_ CD4^+^ and CD8^+^ T lymphocytes with PMA stimulation. Following similar criteria to those employed with S-specific pool stimulation, all of the patients were HRs or NRs according to the percentage and numbers of IFNγ-producing TCM and TEM subpopulations of CD4+ and CD8+ T lymphocytes.

### 3.5. Concordance of Humoral and SARS-CoV-2-Specific T Lymphocyte Memory Response to the mRNA Vaccines

**Figure 4:**
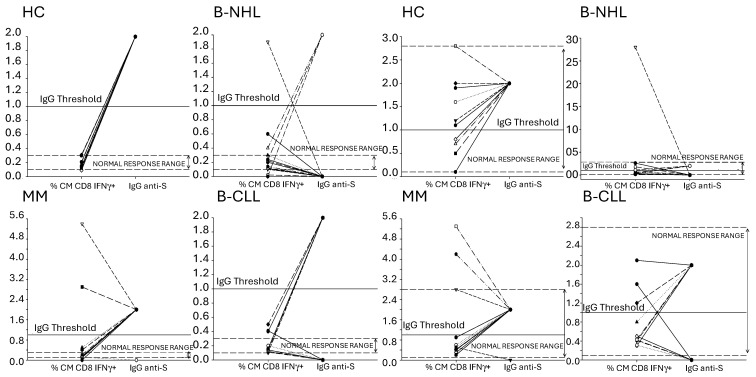
Concordance between the anti-SARS-CoV-2 protein S IgG antibody and the percentage of IFNγ-producing T_CM_ subpopulation of CD8^+^ T lymphocytes. The graphs represent the percentage of IFNγ-producing T_CM_ subpopulation of CD8^+^ T lymphocytes and the production or not of anti-S IgG log AU/mL.

An optimal coordinated humoral and cellular response is essential for the effective prevention and control of COVID-19. Thus, the HCs and patients were subdivided into two groups according to the presence of IgG antibodies to SARS-CoV-2: those showing a non-reactive response (index < 1) were considered humoral response negative, and those showing a reactive response (index ≥ 1.0) were considered humoral response positive. Therefore, in Figure 4, we defined the threshold for IgG antibodies against SARS-CoV-2 at a value of 1.

Thus, all HCs generated a humoral response as well as their T_CM_ subpopulation produced IFNγ after stimulation with SARS-CoV-2 protein S. For this reason, we considered that the HCs had a positive cellular and humoral response (Figure 4, HC). Considering this, we also decided to include in the graphs a range of normal responses, which were defined by the percentage of the cellular response of the HCs.

We found that 52.5% (21/40) of patients exhibited both S-specific humoral as well as CD4^+^ and CD8^+^ T_CM_ lymphocyte response, and 47.5% (19/40) had a discordant response, defined as the absence of an S-specific humoral response in the presence of an S-specific T_CM_ lymphocyte response vaccination. This discordant immune response was mainly observed in patients with B-NHL. Only 12.5% (2/16) and 18.75% (3/16) had a positive CD4^+^ and CD8^+^ cellular and humoral response, respectively; however, 68.75% (11/16) and 100% (16/16) of them elicited a CD4^+^ and CD8^+^ T_CM_ lymphocyte response, respectively (Figure 4 B-NHL). This was mainly due to a worse humoral response in B-NHL patients receiving anti-CD20 based therapy. Regarding the MM patients, 92.3% (12/13) generated both a T_CM_ (CD4^+^ and CD8^+^) and humoral response (Figure 4, MM). Finally, 54.5% (6/11) of patients with B-CLL had a positive T_CM_ CD4^+^ and CD8^+^ and humoral responses, while the other patients (5/11) had only a positive T_CM_ response (Figure 4, B-CLL).

### 3.6. SARS-CoV-2 Breakthrough Infection in Vaccinated BCMs

During follow-up, a median of 165 days after the second immunization until the third dose [IQR 137–172], two (5%) out of the 40 fully vaccinated (two doses) patients with B-NHL had a breakthrough infection and were rapidly treated with Remdesivir as part of our institutional protocol. Both patients required ICU admission and one of them died from COVID-19 pneumonitis. The median time to breakthrough infection was 84 days (range 81–87) from completion of vaccination.

## 4. Discussion

Until now this is the largest study to provide a comprehensive and deep analysis of humoral response characterization, the T-lymphocyte response, and memory profile after two administrations of the SARS-CoV-2 mRNA vaccine in BCMs under active hematological treatment. We found spike-specific CD4^+^ and CD8^+^ responses in 90% and 100% of the vaccinated patients, respectively, irrespective of any observed attenuation in humoral immunity. Our study found that CD4^+^ and CD8^+^ T lymphocyte responses after mRNA vaccination were composed predominantly of T_CM_ and T_EM_ lymphocytes in both the BCM patients and HCs. This is important since a stable memory pool could effectively protect against potential future SARS-CoV-2 infections by their rapid recruitment in the immune response.

Among the 40 vaccinated BCMs patients included in this study, we observed a highly variable magnitude of T-lymphocyte responses to SARS-CoV-2 spike peptides. This is in line with other publications showing that COVID-19 mRNA vaccines elicit a strong and robust cellular response to the viral spike proteins in patients with different hematologic malignancies [21,22,23].

Our most interesting finding was that about 30% of our BCM patients on active therapy showed significantly high frequencies of IFN-γ-producing S-specific TCM and TEM CD4^+^ and CD8^+^ T lymphocytes (HR patients). Various mechanisms that are not mutually exclusive may be involved in this marked responsiveness of the T lymphocytes in this population of BCM patients. First, a potential increased antigen presenting cell-mediated T-cell activation may be suggested. It has been postulated that the presence of more activated monocyte-derived antigens presenting lymphocytes at the time of vaccination could be a result of B-lymphocyte depletion [24]. Additionally, immunomodulatory drugs (IMiDS), a common therapy in MM patients, enhance dendritic cell (DC) antigen presentation, resulting in the activation of CD4^+^ and CD8^+^ T lymphocytes and increased IFN-γ production [25].

Second, an increase in the capacity of memory CD4^+^ and CD8^+^ T lymphocytes in BCM patients might facilitate better responsiveness to the vaccine, reflected by the higher frequency of S-peptide-specific T cells. Of note was the difference in the distribution of activation subsets of CD4^+^ and CD8^+^ T lymphocytes between patients and the HCs in samples without stimulation (basal level). The frequency of CD4^+^ and CD8^+^ T_CM_ and T_EM_ lymphocytes was higher in BCM patients compared to the HCs. These findings are consistent with previous reports [26,27,28] and may indicate a better trend in these patients toward the activation/differentiation of memory T lymphocytes. Whether this pattern of T-lymphocyte differentiation may relate to the disease per se or to the treatment cannot be fully elucidated at present. It has been shown that B-NHL patients on anti-CD20 antibody monotherapy or anti-CD19 CAR T treatment are able to mount potent S-specific CD4^+^ and CD8^+^ T lymphocyte responses following COVID-19 mRNA vaccination, despite impaired humoral responses [29,30]. Considering previous studies in CLL patients receiving Bruton’s tyrosine kinase (BTK) inhibitors [31,32], CD4^+^ T lymphocytes could exhibit increased reactivity to new antigens such as SARS-CoV peptides because these drugs can restore TCR repertoire diversity and alter the composition of T-lymphocyte subtypes by exerting selective Th1 pressure. Bortezomib, a proteasome inhibitor used in the first-line of MM treatment, enhances the production of IFN-γ and downregulates PD-1 expression in CD8^+^ T lymphocytes [33].

Third, a decrease in suppressive regulatory mechanisms mediated by other cells of the immune system upon activation subsets of memory T lymphocytes may be suggested. Our demonstration that T-cell responses within the B-NHL cohort were increased, despite receiving rituximab-chemotherapy-based regimens (R-CHOP), could potentially be explained by cyclophosphamide-induced Treg depletion [34]. However, we could not investigate these findings in our study. The relevance of regulatory factors in this T-cell activation is highlighted by the fact that the few patients with poor T-cell response had normal IFN-γ expression after non-specific PMA stimulation. Our study provides new information on the t-response to COVID-19 mRNA vaccines in patients treated with cyclophosphamide.

BCM patients were classified as responders and non-responders, taking into account the antibody concentrations 1 month after complete vaccination. T_CM_ and T_EM_ CD4^+^ and CD8^+^ T lymphocyte responses after the second vaccination dose were similar in the two categories (52.5% in seropositive and 47.5% in seronegative patients), which indicates no relationship between the humoral response, more specifically the antibody concentrations, and developed a cellular immune response after vaccination. These findings indicate that the generation of a specific T lymphocyte memory against the SARS-CoV2 S protein is not sufficient to achieve adequate B lymphocyte activation and production of S-specific antibodies. This marked limitation of the capacity to generate specific antibodies against the vaccine antigen is especially manifest in B-NHL. In addition to the B-lymphocyte dysfunction associated with the disease pathogenesis, it can be proposed that anti-CD20 based therapy contributes to the development of this deficient humoral response. The findings of other groups analyzing T-lymphocyte and antibody responses in BCM patients after SARS-CoV-2 vaccination are in accordance with our results [13,35].

Finally, to what extent the magnitude of the memory T-lymphocyte response alone, after infection, or vaccination protects against severe disease in BCM patients on active therapy is unclear. In the present study, 100% of BCM patients showed a marked memory CD8^+^ T cell response to mRNA SARS-CoV-2 vaccines. Because cytotoxic CD8+ T cells comprise the major effector arm in clearing SARS-CoV-2 infections, it is perhaps not surprising that the infections are less serious in active treatment BCM patients. In our series, only two patients (seronegative B-NHL patients) that presented with a breakthrough COVID-19 infection developed severe disease and required ICU admission; one died.

There were limitations to our study. First, we did not assess the kinetics of memory T-lymphocyte responses over time, which is relevant to evaluate the long-term protection and establish its duration. Nonetheless, frequencies of virus-specific memory T lymphocytes have been reported to be stable for several months after vaccination [36]. A second limitation of our study is that it may not reflect the vaccine activity in lymphoid or other tissues as it exclusively focused on cellular responses in circulating T lymphocytes [37,38]. Third, we did not evaluate the neutralizing activity of the antibodies nor the presence of antibodies against the SARS-CoV-2 nucleoprotein. Fourth, the limited number of patients in each cohort did not allow us to address whether hybrid immunity leads to more clonally diverse memory responses. Finally, for the same reason above-mentioned, the humoral and magnitude of T-lymphocyte responses were not stratified by the demographic characteristics and type of anti-cancer treatments, which will be addressed in a future study.

## 5. Conclusions

In conclusion, we demonstrated robust S-reactive CD4^+^ and CD8^+^ T lymphocyte responses with T_CM_ and T_EM_ phenotypes in all BCM patients after two doses of the mRNA vaccine, which could be suggestive of the establishment of long-lived immunity. Although our study showed that B-NHL patients undergoing treatment with the anti-CD20 antibody plus chemotherapy have a depressing low seroconversion rate, the fascinating data on the T-lymphocyte-specific response suggest the establishment of long-lived immunity despite the lack of seroconversion. This finding is important considering the growing evidence of the principal role of the T-lymphocyte response in reducing hospitalization and death by SARS-CoV-2 infection. Further research is crucial to analyze the durability of SARS-CoV-2 cellular immunity levels after multiple booster vaccine doses in these patients at higher risk of severe COVID-19.

## Figures and Tables

**Table 1:** Main baseline demographic, clinical, and laboratory data of the patients (n = 40) and healthy controls (n = 10) included in the study. Abbreviations: IQR—interquartile range, NA—not applicable. † Anti-CD20 monotherapy (7), R-CHOP-like (7), R-Bendamustine (1). Φ antiCD38 + VCD (bortezomib, cyclophosphamide, dexamethasone), (3) * Other target therapies: VCD.

Variable	Entire Cohort (n = 40)	B-NHL (n = 16)	CLL (n = 11)	MM (n = 13)	Control (n = 10)
**Age, yr; median (IQR)**	68 (61–72)	69 (59.8–72)	64 (55.5–74)	68 (63–71)	59 (59–59)
**Male sex; n (%)**	27 (67.5)	8 (50)	8 (72.7)	11 (84.6)	6 (60)
**Previous COVID-19, n (%)**	8 (20)	4 (25)	1 (9)	3 (23)	0
**Treatment, n (%)**					NA
Anti-CD20 +/− chemotherapy †	15 (37.5)	15 (93.8)	0	0	
BTK inhibitors	11 (27.5)	1 (6.3)	10 (90.9)	0	
Anti-CD38 based therapy Φ	7 (17.5)	0	0	7 (53.8)	
IMIDs alone	5 (12.5)	0	0	5 (38.5)	
Bcl-2 inhibitors	1 (2.5)	0	1 (9)	0	
Other target therapies *	1 (2.5)	0	0	1 (7.7)	
**Lines of therapy, n (%)**					NA
1	24 (60)	11 (68.8)	7 (63.6)	6 (46.2)	
2	9 (22.5)	2 (12.5)	2 (18.2)	5 (38.5)	
3 or more	7 (17.5)	3 (18.8)	2 (18.2)	2 (15.4)	
**Laboratory parameters**					
Absolute lymphocyte count, 109/L median (IQR)	1425 (902.5–2795)	1150 (555–1462)	3880 (1655–7280)	1430 (850–1800)	1770 (1542–2218)
IgG, mg/dL median (IQR)	650 (493.25–969)	790.5 (629–969)	665 (568–1235)	485 (443–614)	1130 (1032–1172)
IgM, mg/dL median (IQR)	23 (19–49.25)	27.5 (19–56.2)	25 (19.5–51)	19 (19–34)	70.5 (48.5–109)
IgA, mg/dL median (IQR)	83 (46.25–173.5)	124.5 (75.8–213)	103 (59–194)	41 (28–72)	296 (203–381)

## Data Availability

The raw data supporting the conclusions of this article will be made available by the authors on request if necessary.

## Supplementary Materials

The following supporting information can be downloaded at: https://www.mdpi.com/article/10.3390/vaccines12090961/s1, Figure S1. Study Flowchart. B-NHL, B-lymphocyte Non-Hodgkin B-NHL; CLL, Chronic. Figure S2. Dot plots represent the percentage of intralymphocyteular IFNγ secretion from TCM and TEM subpopulations of CD4+ and CD8+ T after stimulation with the peptide pool, in a HC and a lymphoma patient, in a MM patient and in a B-CLL patient. Figure S3. Percentage and numbers of intralymphocyteular IFNγ secretion from TCM and TEM subpopulations of CD4+ and CD8+ T after stimulation with the peptide pool. A, B Box plots represent the percentage of IFNγ production by the TCM subpopulations of CD4+ and CD8+ T. C, D Box plots represent the percentage of IFNγ production by TEM subpopulations of CD4+ and CD8+ T. E, F Box plots represent the numbers of IFNγ production by TCM subpopulation of CD4+ and CD8+ T G, H Box plots represent the numbers of IFNγ production by TEM subpopulation of CD4+ and CD8+ T HCs, B-NHL, MM, B-CLL. * *p* < 0.05 HCs vs. Patients; † *p* < 0.05 B-NHL vs. MM; α *p* < 0.05 B-NHL vs. B-CLL; β *p* < 0.05 MM vs. B-CLL. Figure S4. Dot plots represent the percentage of intralymphocyteular IFNγ secretion from TCM and TEM subpopulations of CD4+ and CD8+ T after stimulation with PMA, in a HC and a lymphoma patient, in a MM patient and in a B-CLL patient. Figure S5. Percentage and numbers of intralymphocyteular IFNγ secretion from TCM and TEM subpopulations of CD4+ and CD8+ T after stimulation with PMA. A, B Box plots represent the percentage of IFNγ production by the TCM subpopulations of CD4+ and CD8+ T. C, D Box plots represent the percentage of IFNγ production by TEM subpopulations of CD4+ and CD8+ T lymphocyte. E, F Box plots represent the numbers of IFNγ production by TCM subpopulation of CD4+ and CD8+ T. G, H Box plots represent the numbers of IFNγ production by TEM subpopulation of CD4+ and CD8+ T lymphocyte. HCs, B-NHL, MM, B-CLL. * *p* < 0.05 HCs vs. Patients; † *p* < 0.05 B-NHL vs. MM; α *p* < 0.05 B-NHL vs. B-CLL; β *p* < 0.05 MM vs. B-CLL. Lymphocytic Leukemia; MM, Multiple Myeloma; ICF, Informed Consent Form.
