# Supplementary material for: SARS-CoV-2-Specific T Lymphocytes Analysis in mRNA-Vaccinated Patients with B-Cell Lymphoid Malignancies on Active Treatment
Source: Vaccines (Basel). 2024 Aug 26;12(9):961. doi: 10.3390/vaccines12090961 (PMC11435597; doi:10.3390/vaccines12090961)
Supplement: Supplementary file 1 [file vaccines-12-00961-s001.zip › vaccines-3111882-supplementary.pdf]

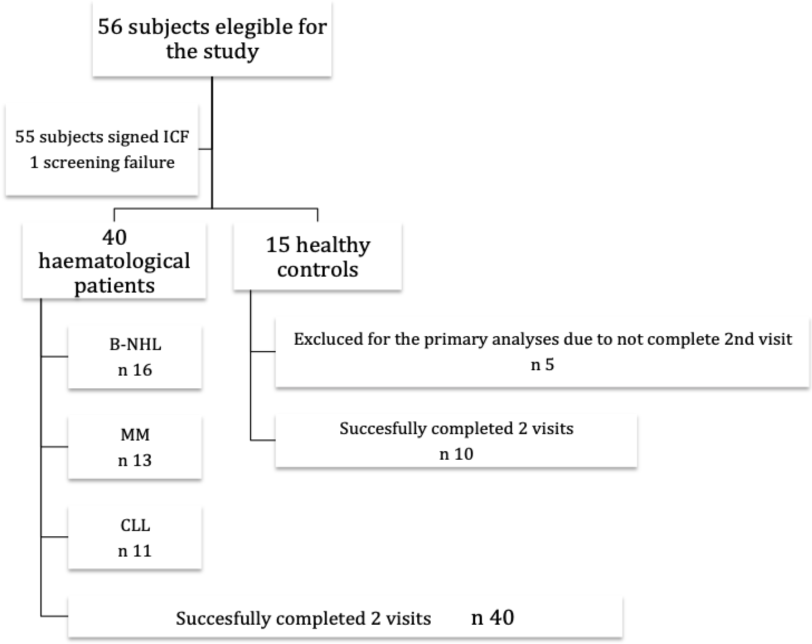


**Supplementary material Figure S1.** Study Flowchart. B-NHL, B-lymphocyte Non-Hodgkin B-NHL; CLL, Chronic Lymphocytic Leukemia; MM, Multiple Myeloma; ICF, Informed Consent Form.

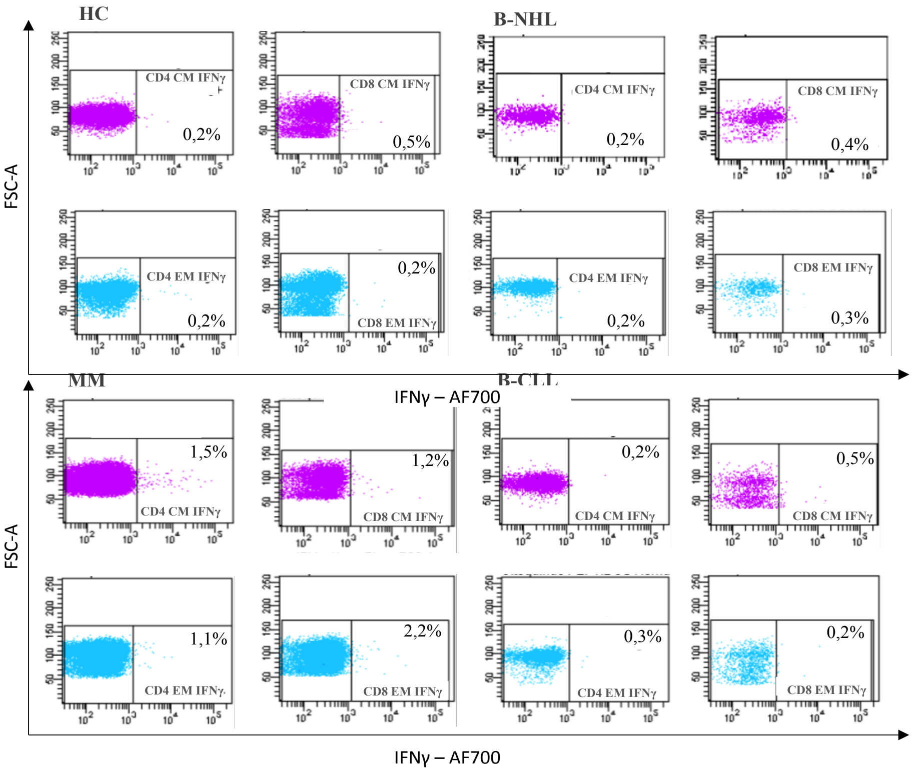


**Supplementary material Figure S2.** Dot plots represent the percentage of intralymphocyteular IFNγ secretion from $T_{CM}$ and $T_{EM}$ subpopulations of $CD4^+$ and $CD8^+$ T after stimulation with the peptide pool, in a HC and a lymphoma patient, in a MM patient and in a B-CLL patient.

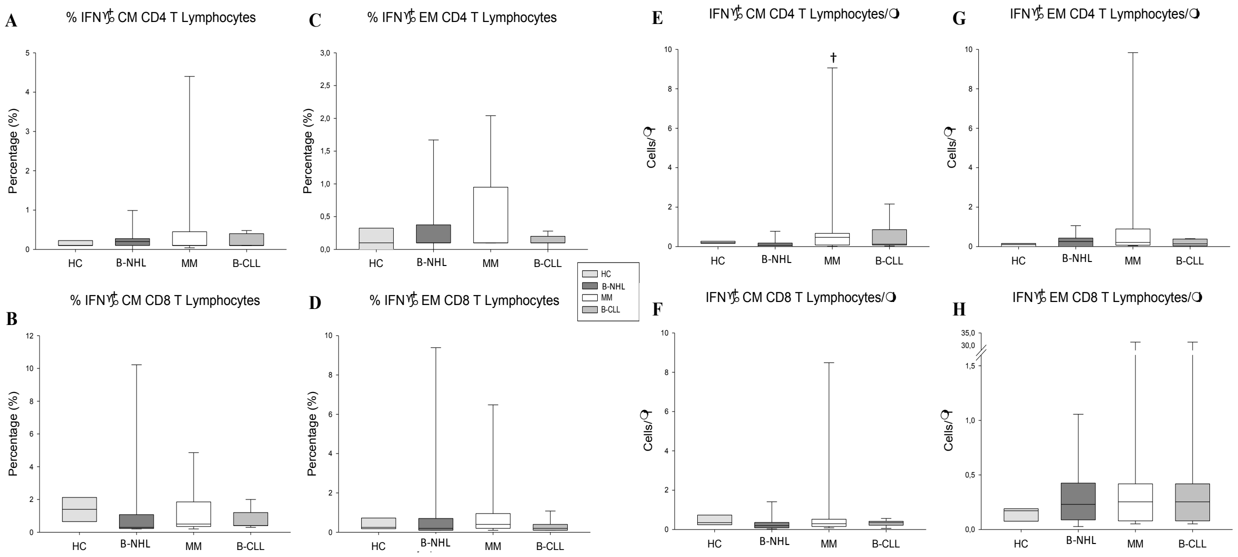


**Supplementary material Figure S3. Percentage and numbers of intralymphocyteular IFNγ secretion from $T_{CM}$ and $T_{EM}$ subpopulations of CD4$^+$ and CD8$^+$ T after stimulation with the peptide pool. A, B** Box plots represent the percentage of IFNγ production by the $T_{CM}$ subpopulations of CD4$^+$ and CD8$^+$ T. **C, D** Box plots represent the percentage of IFNγ production by $T_{EM}$ subpopulations of CD4$^+$ and CD8$^+$ T. **E, F** Box plots represent the numbers of IFNγ production by $T_{CM}$ subpopulation of CD4$^+$ and CD8$^+$ T **G, H** Box plots represent the numbers of IFNγ production by $T_{EM}$ subpopulation of CD4$^+$ and CD8$^+$ T HCs, B-NHL, MM, B-CLL. * $p<0.05$ HCs vs Patients; † $p<0.05$ B-NHL vs MM; α $p<0.05$ B-NHL vs B-CLL; β $p<0.05$ MM vs B-CLL.

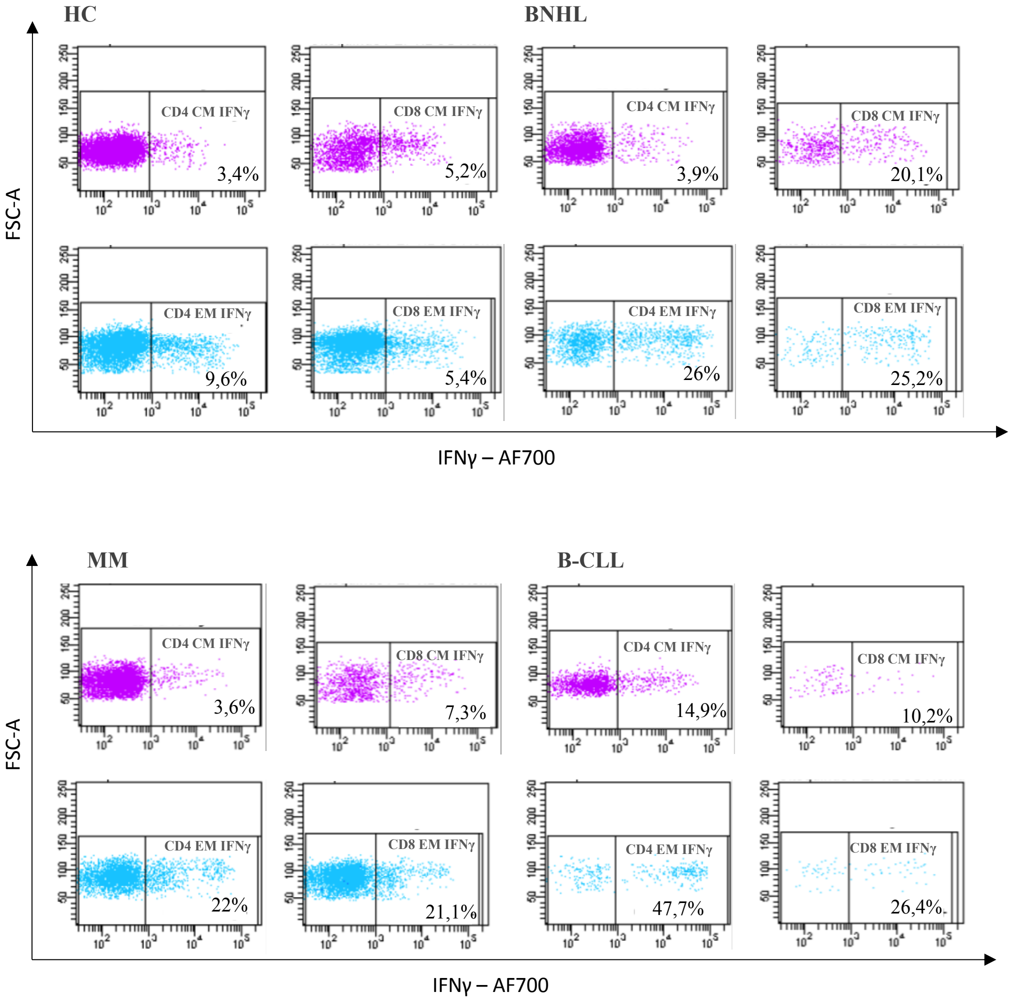


**Supplementary material Figure S4.** Dot plots represent the percentage of intralymphocyteular IFNγ secretion from $T_{CM}$ and $T_{EM}$ subpopulations of CD4$^+$ and CD8$^+$ T after stimulation with PMA, in a HC and a lymphoma patient, in a MM patient and in a B-CLL patient.

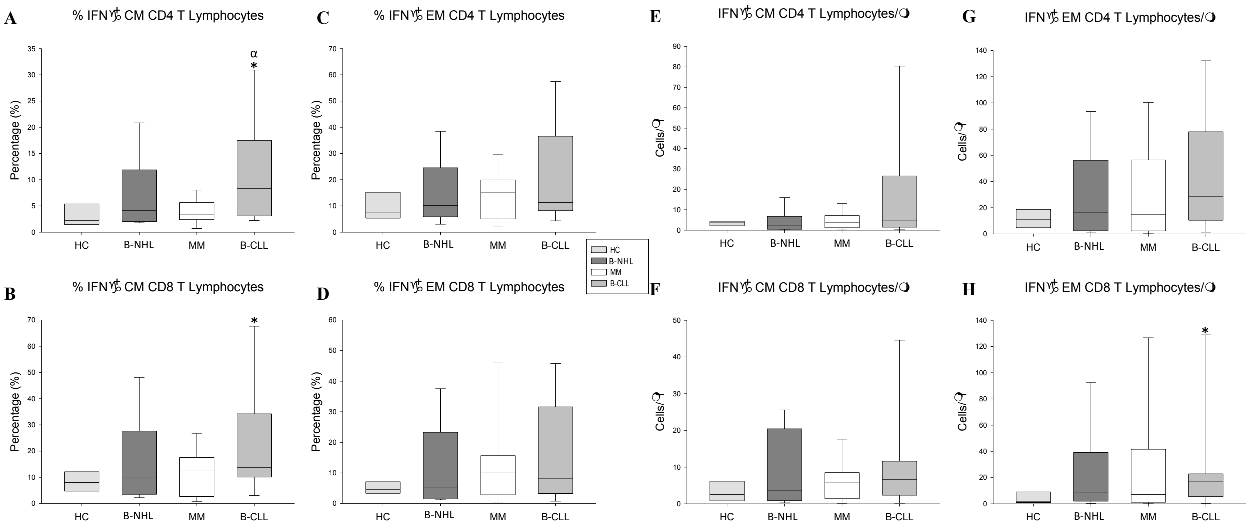


**Supplementary material Figure S5. Percentage and numbers of intralymphocyteular IFNγ secretion from $T_{CM}$ and $T_{EM}$ subpopulations of $CD4^+$ and $CD8^+$ T after stimulation with PMA. A, B** Box plots represent the percentage of IFNγ production by the $T_{CM}$ subpopulations of $CD4^+$ and $CD8^+$ T. **C, D** Box plots represent the percentage of IFNγ production by $T_{EM}$ subpopulations of $CD4^+$ and $CD8^+$ T lymphocyte. **E, F** Box plots represent the numbers of IFNγ production by $T_{CM}$ subpopulation of $CD4^+$ and $CD8^+$ T. **G, H** Box plots represent the numbers of IFNγ production by $T_{EM}$ subpopulation of $CD4^+$ and $CD8^+$ T lymphocyte. HCs, B-NHL, MM, B-CLL. * $p<0.05$ HCs vs Patients; † $p<0.05$ B-NHL vs MM; α $p<0.05$ B-NHL vs B-CLL; β $p<0.05$ MM vs B-CLL.
